# Author Correction: High-resolution annotation of the mouse preimplantation embryo transcriptome using long-read sequencing

**DOI:** 10.1038/s41467-021-22148-6

**Published:** 2021-03-15

**Authors:** Yunbo Qiao, Chao Ren, Shisheng Huang, Jie Yuan, Xingchen Liu, Jiao Fan, Jianxiang Lin, Susu Wu, Qiuzhen Chen, Xiaochen Bo, Xiangyang Li, Xingxu Huang, Zhen Liu, Wenjie Shu

**Affiliations:** 1 Precise Genome Engineering Center, School of Life Sciences, Guangzhou University, 510006 Guangzhou, China; 2 Department of Biotechnology, Beijing Institute of Radiation Medicine, 100850 Beijing, China; 3 School of Life Science and Technology, ShanghaiTech University, 201210 Shanghai, China; 4 University of Chinese Academy of Sciences, 100049 Beijing, China; 5 Institute of Neuroscience, Key Laboratory of Primate Neurobiology, CAS Center for Excellence in Brain Science and Intelligence Technology, Chinese Academy of Sciences, 200031 Shanghai, China; 6 Institute of Geriatrics & National Clinical Research Center of Geriatrics Disease, 2nd Medical Center of Chinese PLA General Hospital, 100853 Beijing, China; 7 Computer School, University of South China, 421001 Hengyang, China

**Keywords:** Next-generation sequencing, RNA splicing, Transcriptomics, Embryonic germ cells

Correction to: *Nature Communications* 10.1038/s41467-020-16444-w, published online 27 May 2020.

The original version of this Article contained an error in the author affiliations. The affiliation of Xingchen Liu with 4: University of Chinese Academy of Sciences, 100049 Beijing, China was inadvertently omitted.

This has now been corrected in both the PDF and HTML versions of the Article.

