# Supplementary material for: High-resolution annotation of the mouse preimplantation embryo transcriptome using long-read sequencing
Source: Nat Commun. 2020 May 27;11:2653. doi: 10.1038/s41467-020-16444-w (PMC7253418; doi:10.1038/s41467-020-16444-w)
Supplement: Supplementary file 1 — Supplementary Information [file 41467_2020_16444_MOESM1_ESM.pdf]

Supplementary Information for

High resolution annotation of preimplantation embryo transcriptome using long-read sequencing

Qiao et al.

a

Short-read sequencing quality control

Samples	Total sequences	Read length (pair-end)	Q30	CG percent
Sperm_1	31637904	150	88.53	47.88
Sperm_2	33692189	150	91.48	49.70
Oocyte_1	28534912	150	88.23	46.74
Oocyte_2	27147848	150	89.81	48.60
1-cell_1	19105432	150	86.34	48.68
1-cell_2	38851666	150	90.52	47.79
2-cell_1	19637095	150	88.56	49.09
2-cell_2	45527417	150	87.88	45.92
4-cell_1	21542325	150	88.54	46.44
4-cell_2	24746576	150	88.80	46.82
8-cell_1	30758439	150	91.94	49.97
8-cell_2	27344364	150	88.67	47.57
Blastocyst_1	24220851	150	89.83	48.03
Blastocyst_2	28223489	150	90.24	51.84

b

Long-read sequencing quality control

Samples	Polymerase reads	Polymerase reads N50	Subreads	Mean length of subreads	CCS	Insert Size
Sperm	420225	73273	18811197	818	364680	2150
Oocyte	343546	83822	14420594	773	236135	5143
1-cell_seq1	93751	64847	4229950	574	62549	4382
1-cell_seq2	303121	41126	7474332	636	186173	3293
2-cell	234699	76126	9030981	1062	202994	2442
4-cell	247871	79612	9733923	1159	223950	2185
8-cell	319045	87637	15443002	1016	292558	1942
Blastocyst_seq1	155615	76455	7393076	732	126881	2518
Blastocyst_seq2	228246	74756	9235454	872	184441	2961

c

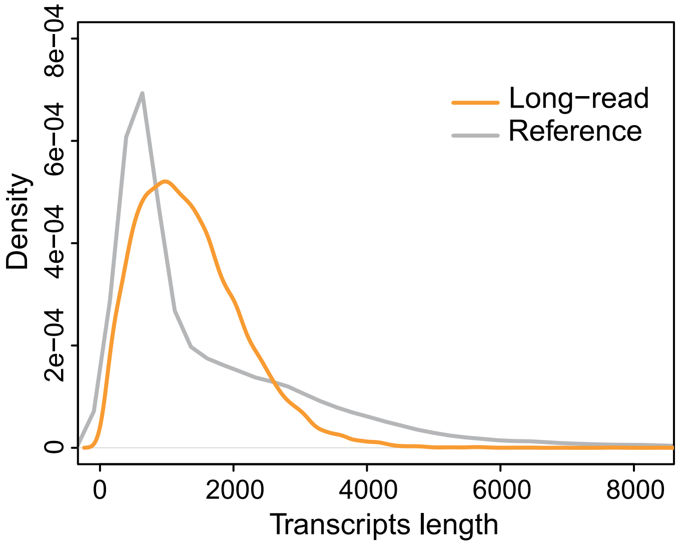

Supplementary Figure 1

Supplementary Figure 1. Quality control of sequencing data in this study. **(a)** Quality control for short-read sequencing data. Each sample contains two replicates ("_1" and "_2"). **(b)** Quality control for long-read sequencing data. For 1-cell and blastocyst, sequencing replicates but not biological replicates were performed to generate enough sequencing reads. These two files were integrated together for analysis. **(c)** Comparison of the transcript length of long-read and GENCODE annotation.

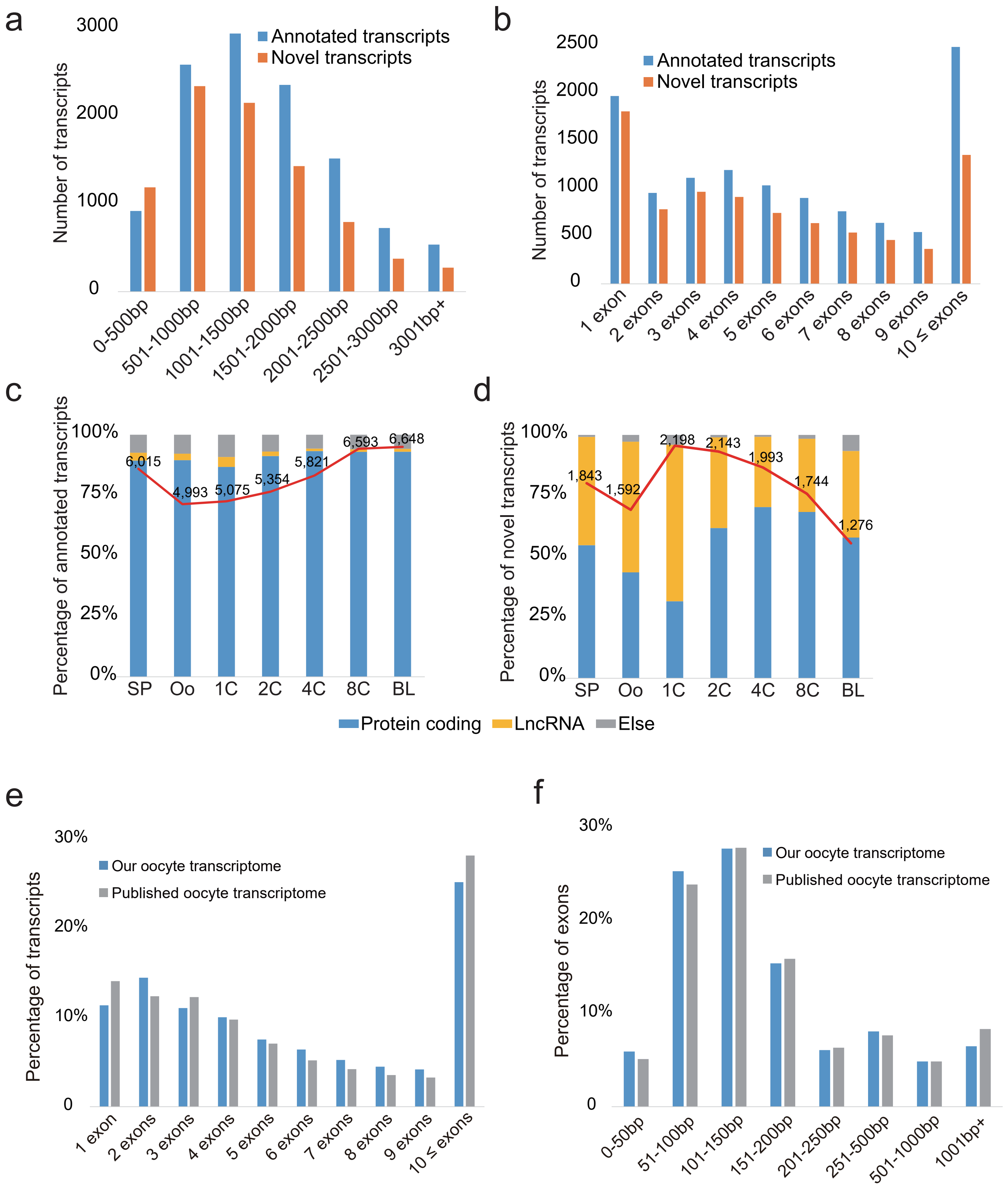

Supplementary Figure 2

Supplementary Figure 2. Characterizing the long-read transcripts.

(a) The length distributions of annotated and novel transcripts identified by long-read data. **(b)** The exon count distributions of annotated and novel transcripts identified by long-read data. **(c)** Classification of annotated transcripts in seven stages according to GENCODE annotation. Bar plot represents the percentage of transcripts in each category and the red line represents the total number of annotated transcripts in each stage. **(d)** Classification of novel transcripts in seven stages based on protein-coding potential and the length of transcripts. Bar plot represents the percentage of transcripts in each category and red line represents the total number of novel transcripts in each stage. **(e)** The exon count distributions of transcripts identified by our data and a published data in oocyte[1]. **(f)** The exon length distributions of transcripts identified by our data and a published data in oocyte[1].

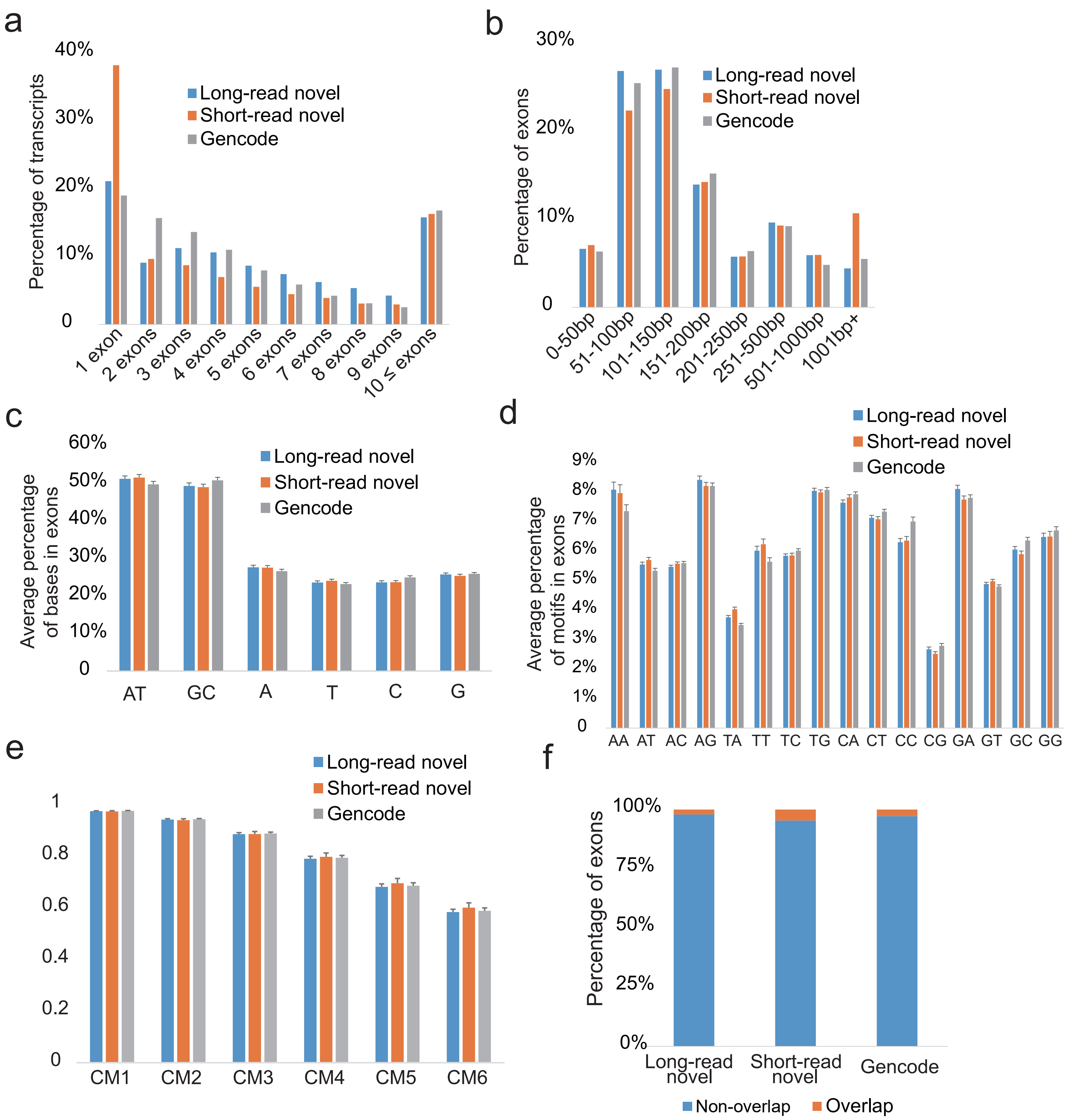

Supplementary Figure 3

Supplementary Figure 3. Characterization of transcripts identified by long-read data and short-read data.

(a) The exon count distributions of novel transcripts identified by long-read data, short-read data, and transcripts from GENCODE annotation. **(b)** The exon length distributions of novel transcripts identified by long-read data, short-read data, and transcripts from GENCODE annotation. **(c)** Average percentage of bases in exons of novel transcripts identified by long-read data, short-read data, and transcripts from GENCODE annotation. Data are presented as mean value + S.D. 46263,157647,818415 exons from long-read data, short-read data and GENCODE were used in analysis. **(d)** Average percentage of motifs in exons of novel transcripts identified by long-read data, short-read data, and transcripts from GENCODE annotation. Data are presented as mean value + S.D. 46263,157647,818415 exons from long-read data, short-read data and GENCODE were used in analysis. **(e)** Complexity of Markov model values in exons of novel transcripts identified by long-read data, short-read data, and transcripts from GENCODE annotation. Data are presented as mean value + S.D. 46263,157647,818415 exons from long-read data, short-read data and GENCODE were used in analysis. **(f)** We compared the exons of novel transcripts only identified by long-read data, short-read data, and GENCODE annotation with low complexity regions obtained from RepeatMasker in UCSC table browser respectively, and the overlapping ratio was shown in the histogram.

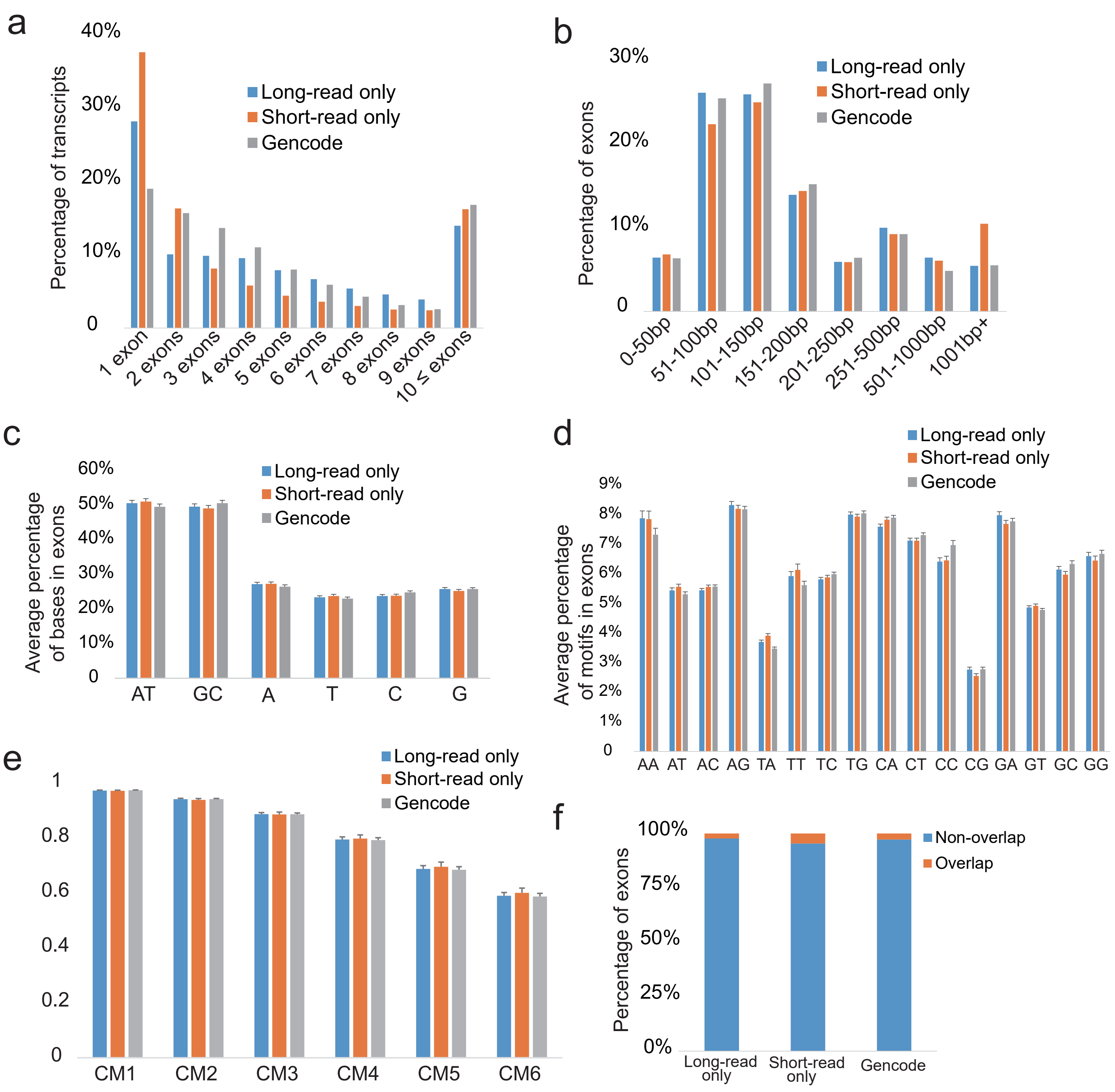

Supplementary Figure 4

Supplementary Figure 4. Characterization of transcripts only identified by long-read data and short-read data.

(a) The exon count distributions of transcripts only identified by long-read data, short-read data, and transcripts from GENCODE annotation. **(b)** The exon length distributions of transcripts only identified by long-read data, short-read data, and transcripts from GENCODE annotation. **(c)** Average percentage of bases in exons of transcripts only identified bylong-read data, short-read data, and transcripts from GENCODE annotation. Data are presented as mean value + S.D. 58497,299072,818415 exons from long-read data, short-read data and GENCODE were used in analysis. **(d)** Average percentage of motifs in exons of transcripts only identified by long-read data, short-read data, and transcripts from GENCODE annotation. Data are presented as mean value + S.D. 58497,299072,818415 exons from long-read data, short-read data and GENCODE were used in analysis. **(e)** Complexity of Markov model values in in exons of transcripts only identified by long-read data, short-read data, and transcripts from GENCODE annotation. Data are presented as mean value + S.D. 58497,299072,818415 exons from long-read data, short-read data and GENCODE were used in analysis. **(f)** We compared the exons of transcripts only identified by long-read data, short-read data, and GENCODE annotation with low complexity regions obtained from RepeatMasker in UCSC table browser respectively, and the overlapping proportion was shown in the histogram.

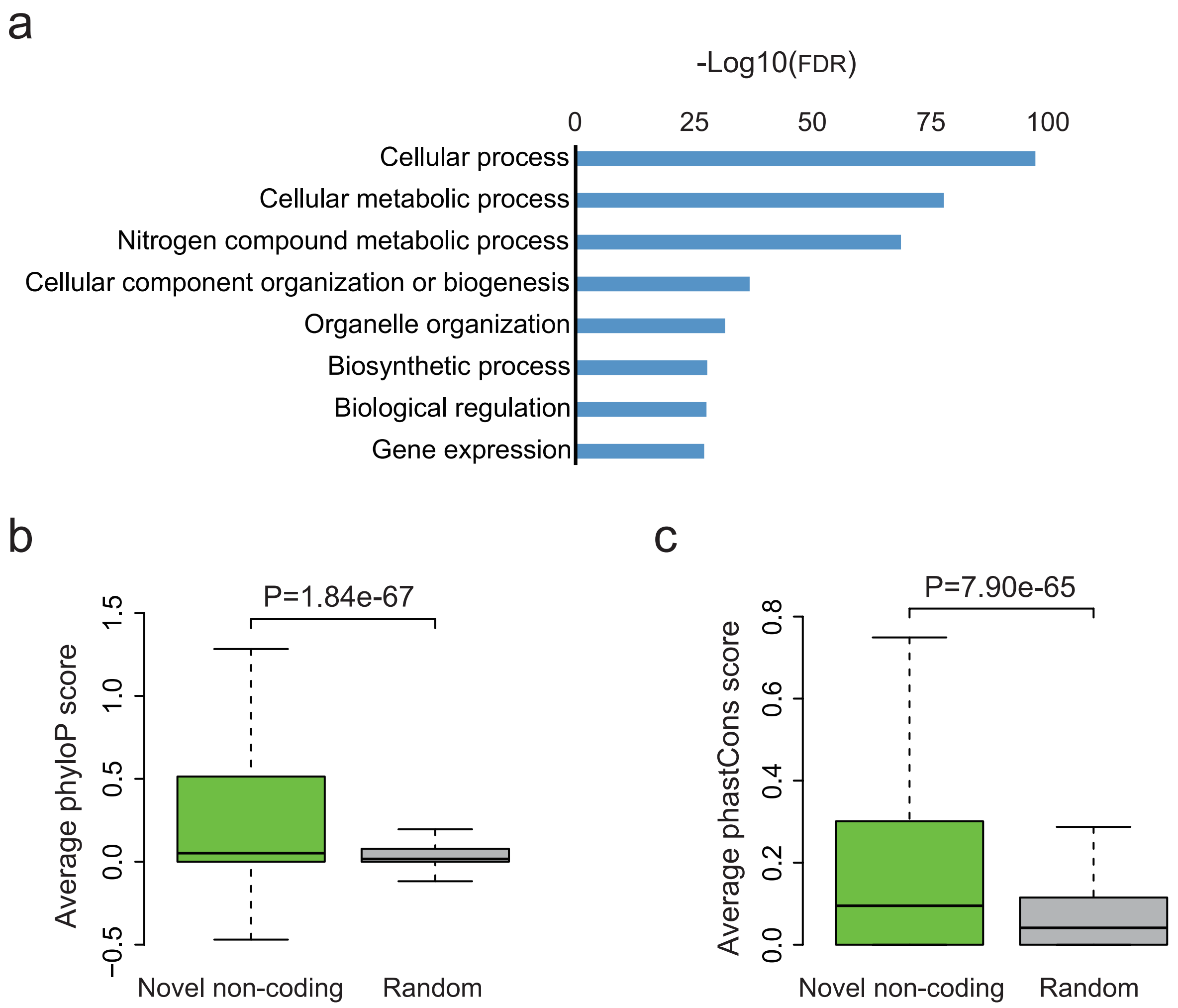

Supplementary Figure 5

Supplementary Figure 5. Functional characterization of novel transcripts. **(a)** The GO enrichment analysis in biological processes for novel coding transcripts predicted by annotated proteins. **(b-c)** Comparison of the average phyloP score and phastCons score of novel noncoding transcripts with sequences in random control regions. P values are calculated from two-tailed Wilcoxon rank sum tests. The boxplots were drawn where the middle line is the median, the lower and upper hinges of box correspond to the first and third quartiles, the lower edge and upper edge correspond to the smallest value and largest value. 3,880 novel noncoding transcripts were calculated in two box plots.

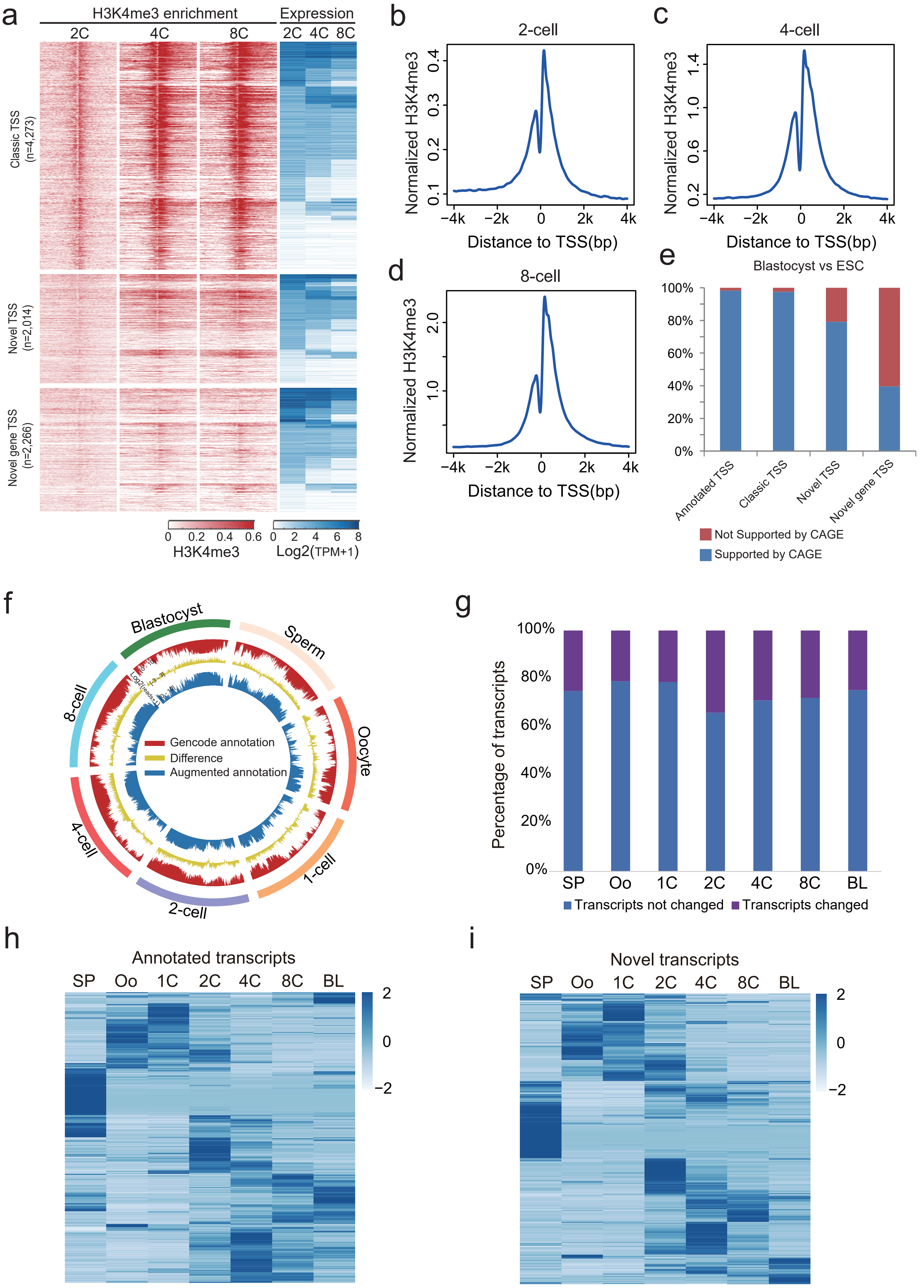

Supplementary Figure 6

Supplementary Figure 6. Validation of annotated and novel transcripts.

(a) Association of H3K4me3 enrichment with gene expression in the identified transcripts. Red heatmaps represent the distribution of the H3K4me3 signals in the promoters of classic TSSs (novel transcripts sharing same TSSs with annotated transcripts), novel TSSs (novel TSSs of novel transcripts within annotated loci), and novel gene TSSs in the 2-cell, 4-cell, and 8-cell stages. Blue heatmaps represent the expression of the corresponding transcripts for these TSSs. **(b-d)** Profiles of the average H3K4me3 signal density on promoters of annotated TSSs in 2-cell, 4-cell, and 8-cell (defined in Methods section). **(e)** CAGE data from mouse ESCs[2] were analyzed to compare with the transcripts with annotated TSSs, classic TSSs, high-confidence novel TSSs of novel splicing isoforms, and high-confidence TSS of novel genes. The blue bars represent TSSs supported by CAGE peaks, and red bars represent TSSs not supported by CAGE peaks. **(f)** Circos plot showing the expression levels of differentially expressed transcripts ($\delta > 5$) annotated when mapping short-read data to genome with GENCODE annotation and GENCODE-augmented annotation as references separately. Red: the expression level of differentially expressed transcripts based on the GENCODE reference. Blue: the expression level of differentially expressed transcripts based on the augmented reference (combination of long-read sequencing and GENCODE). Yellow: the altered expression of differentially expressed transcripts annotated according to the two references. **(g)** The expression of GENCODE-annotated transcripts possessing potential novel splicing isoforms (from long-seq data) was compared by using GENCODE annotation and augmented annotation as reference separately. δ represents the absolute difference value for a transcript from two annotations. Bar plot showed the proportions of transcripts changed ($\delta > 5$) and transcripts not changed ($\delta \leq 5$). **(h-i)** Heatmaps showing the expression of annotated (h) and novel (i) transcripts expression in seven stages. Heatmap shows Z-scores of TPM by row.

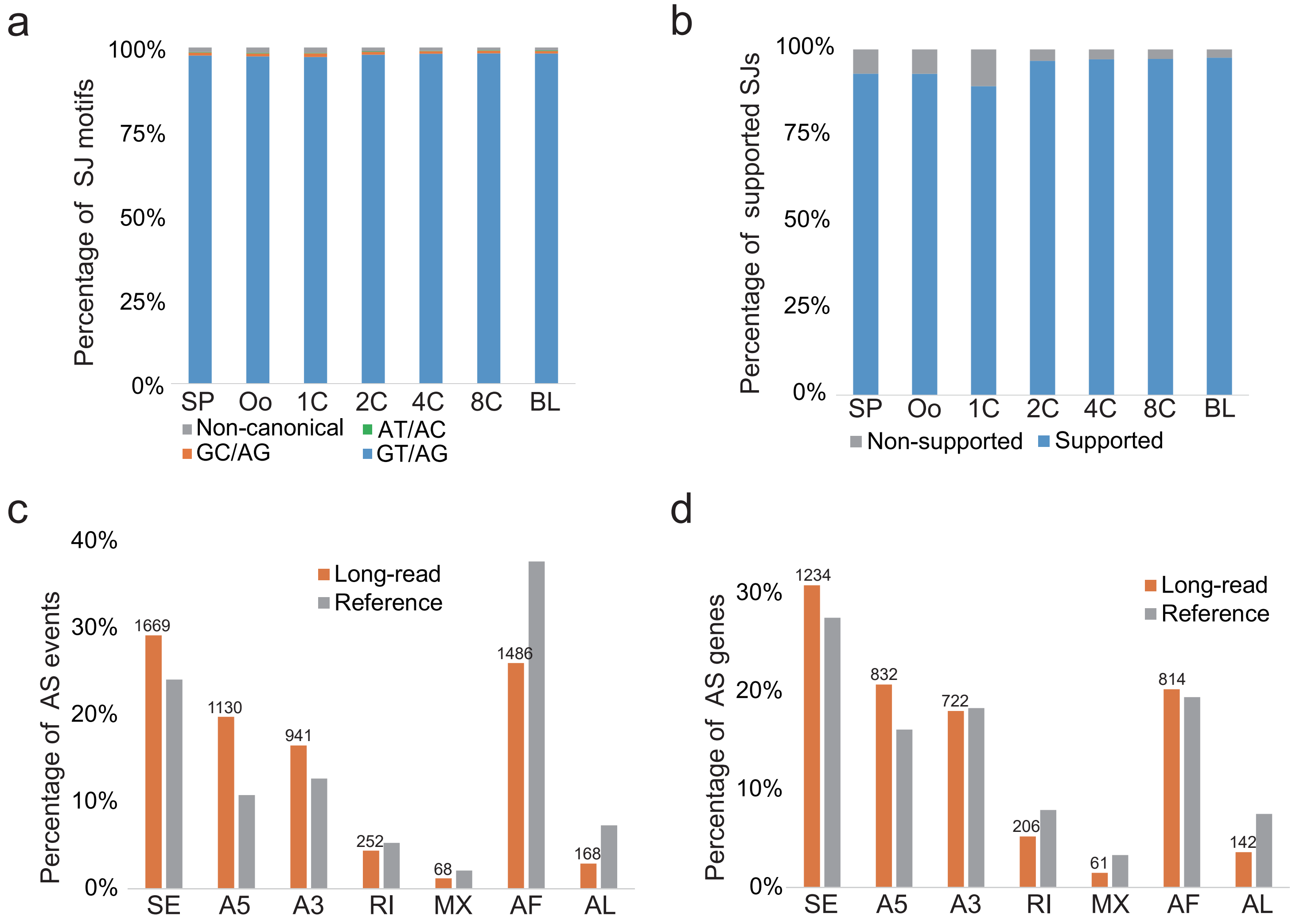

Supplementary Figure 7

Supplementary Figure 7. Characterization of AS by long-read data.

(a) Motif distributions of splicing junction identified by using long-read sequencing in seven stages. **(b)** Verification of long-read transcript junctions by short-read splicing junctions. **(c-d)** Distribution of AS events (c) and genes (d) identified from merged long-read transcripts and GENCODE annotation. The number in the top of orange bars represents the number of AS events (c) and genes (d) identified from merged long-read transcripts.

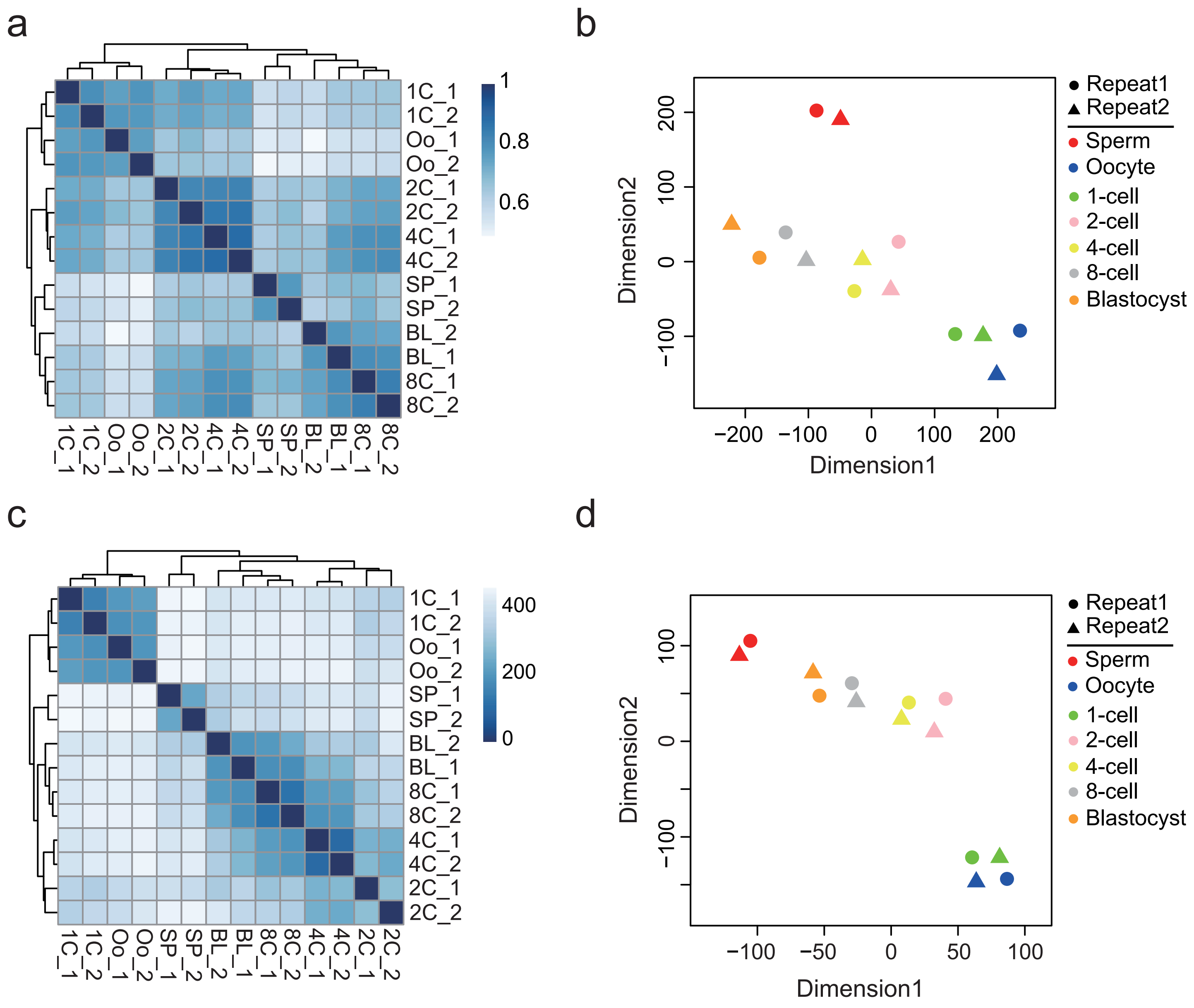

Supplementary Figure 8

Supplementary Figure 8. Clustering analysis of preimplantation embryo development.

(a) Clustering of all samples based on splicing events by long-read data.

(b) Overview of total samples as a tSNE visualization based on splicing.

(c) Clustering of all samples based on gene expression by short-read data.

(d) Overview of total samples as a tSNE visualization based on gene expression.

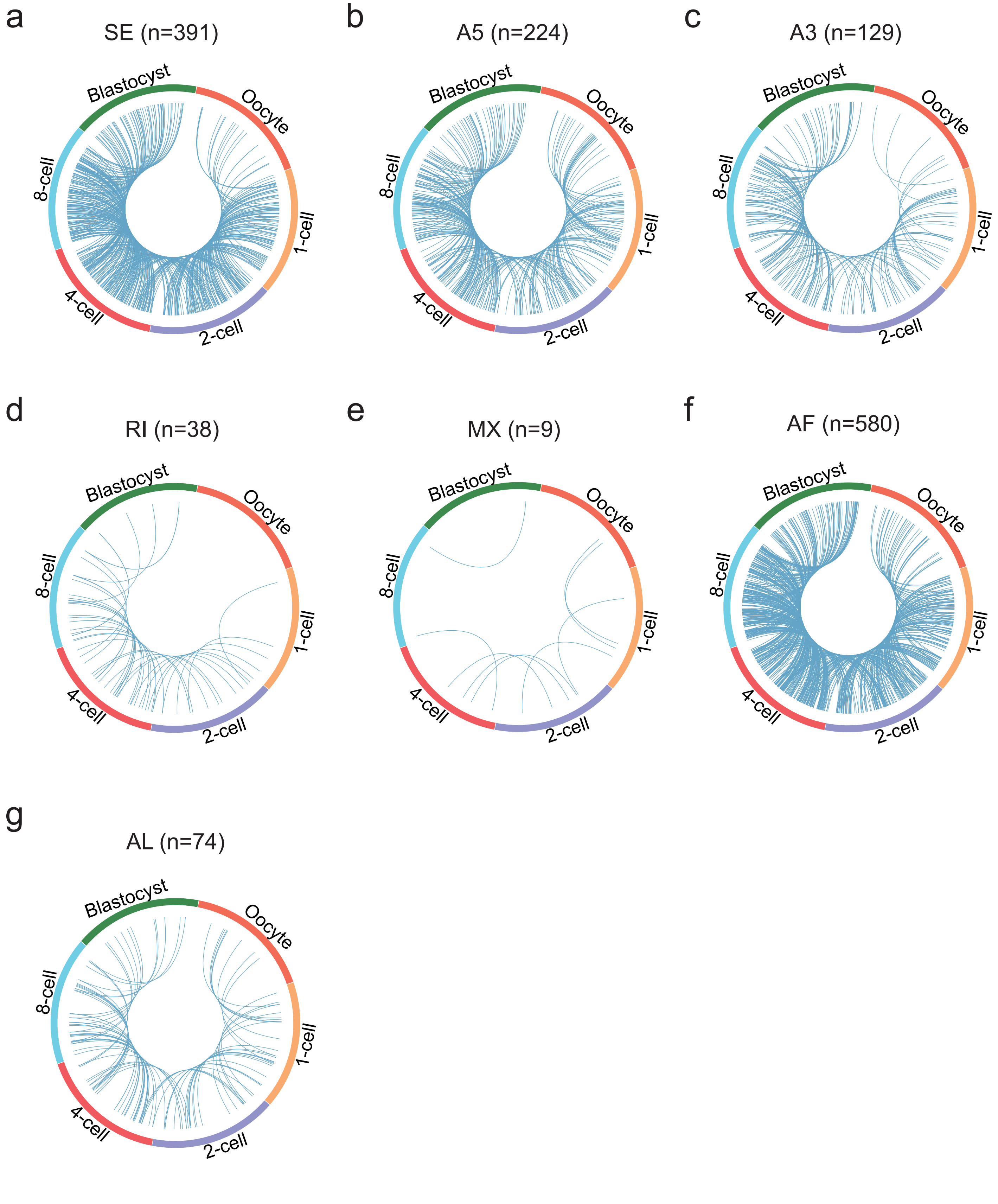

Supplementary Figure 10

Supplementary Figure 9. Dynamic differential splicing events.

(a-g) Overview of differential splicing events of seven types identified in consecutive stages. The blue lines that connect two stages represent the differential splicing events identified in the two stages.

Supplementary Table 1: *P*-values of Fisher-exact test on the number of DAS events between consecutive groups.

	**1-cell_2-cell → 2-cell_4-cell**	**2-cell_4-cell → 4-cell_8-cell**	**4-cell_8-cell→ 8-cell_blastocyst**
Up	0.0003587	1.87E-34	3.72E-17
Down	0.0001029	1.52E-12	1.52E-12

Supplementary Table 2: GO enrichment analysis of the genes associated with these significant DAS events.

GO biological process complete	Mus musculus - REFLIST (22296)	upload_1 (255)	upload_1 (expected)	upload_1 (over/under)	upload_1 (fold Enrichment)	upload_1 (raw P-value)	upload_1 (FDR)
cellular metabolic process (GO:0044237)	6339	131	72.5	+	1.81	2.55E-14	4.02E-10
nitrogen compound metabolic process (GO:0006807)	5705	114	65.25	+	1.75	7.15E-11	2.82E-07
gene expression (GO:0010467)	1591	47	18.2	+	2.58	2.84E-09	4.98E-06
nucleic acid metabolic process (GO:0090304)	1740	49	19.9	+	2.46	6.23E-09	9.83E-06
RNA metabolic process (GO:0016070)	1222	36	13.98	+	2.58	2.82E-07	3.18E-04
G protein-coupled receptor signaling pathway (GO:0007186)	1830	3	20.93	-	0.14	1.42E-06	1.24E-03
RNA processing (GO:0006396)	742	25	8.49	+	2.95	2.30E-06	1.81E-03
cellular component biogenesis (GO:0044085)	2406	52	27.52	+	1.89	1.01E-05	6.39E-03

Supplementary Table 3: sgRNA sequences.

**Name**	**Protospacer sequence**	**PAM**	**Target gene**
sgKdm4dl-1	GAGCAAGAAGAGGATGCCCT	GGG	Kdm4dl
sgKdm4dl-2	GATCACGACATAGTGATTGA	GGG	Kdm4dl
sgXLOC_004958-1	GCTGAGTTCTGTGGCCTGAG	TGG	XLOC_004958
sgXLOC_004958-2	GTCAGAAAGCTATGACGGCG	GGG	XLOC_004958

Supplementary Table 4: PCR primer sequences.

Primer Name	Primer Sequences
TCONS_00001315-F	TGATGTATGGAGGGAGTATG
TCONS_00004813-F	CTCTGTATATCAACCTCAGCC
TCONS_00022541-F	AAGAAGCTGACTTCTCCCCTG
TCONS_00004118-F	CTAGACCTTAGCTTATTCGC
TCONS_00005831-F	GGAGTGTGCTCCCTCTGATT
TCONS_00017425-F	GTTCCTCATGGTCAGGCTAG
TCONS_00006055-F	GAACTACATCAGGGATCTGG
TCONS_00009513-F	CTGCTGGCTACAGTCATGAC
TCONS_00000891-F	CCTTGGATTTTGTCCAGAATGAC
TCONS_00022490-F	CAATGATGACGACTACGAGG
TCONS_00015874-F	GCAAGCCAGTAAGGAACATCT
TCONS_00020867-F	CTAAAGCTTGTGTCTTCCTCG
TCONS_00005014-F	TGGAAGTCTCGTTTGGGCTC
TCONS_00009726-F	ACAAACTGGTGCGGAAGATC
TCONS_00004839-F	TGCTTCCAAAGCATCCGCTC
TCONS_00015595-F	CTTATTGGCCAACAGCATATC
TCONS_00009279-F	CAGCGCAACTTCTGTTCCAC
TCONS_00001969-F	ACGAATGGTTGGGTCACCAG
TCONS_00017927-F	TAGCTGCCTAGAAGGCAGTC
TCONS_00014103-F	GACCACTACACATAGGCTTC
TCONS_00001315-R	CTACGGATTCACGCCTCTC
TCONS_00004813-R	CGAGTCAAGAGAAAGGTTCTC
TCONS_00022541-R	CCCAGAATCCCCAAAGCAATATC
TCONS_00004118-R	GAGGCAGATGGACAGGAAG
TCONS_00005831-R	TCTACAGCTGCTACTGACCA
TCONS_00017425-R	GAAACCACAAGTTCTAAGGG
TCONS_00006055-R	CACCGTAAGTTGTTCTTTGGC
TCONS_00009513-R	GAAGACTTCAGCTTCTTCAGC
TCONS_00000891-R	CCAAGATGGAGGAGGACAG
TCONS_00022490-R	AGAACAGGTTTCTGGGCTAC
TCONS_00015874-R	CTGTACACTGGAGATGTAGG
TCONS_00020867-R	CCTGCTTTGTGACCTGCTTA
TCONS_00005014-R	AGGCCCAGCAGAAGTTAGTG
TCONS_00009726-R	GCTCCATGCTTTGTTCCCAG
TCONS_00004839-R	CTGGATGCTGCCATGCTCTA
TCONS_00015595-R	GAACCATTGTTGGGAATTCGG
TCONS_00009279-R	CTTCCATTCACAGCTGCGAT
TCONS_00001969-R	GTTCAGGTTAGGAGTGTAAGG
TCONS_00017927-R	GAACTACACTGTACAGGAGC
TCONS_00014103-R	AGGATGAGTTTCCCGTCAAC

Kdm4dl-qpcr-F	TTGCATTGGTGCTGGGAAAC
Kdm4dl-qpcr-R	CTCTCTCTGTGTAAGCTTCC
Kdm4dl-RT-F	ACAGGAAGAGCAAGTCCTCC
Kdm4dl-RT-R	AGCCTGCCTTTCCACTCCT
Kdm4dl-GT-F	TTCCCATACAGTGACCAAGG
Kdm4dl-GT-R	TAGCCATAGGGAAAGGTGAC
XLOC_004958-qpcr-F	TGCCGACTTTTTGGCAGGTG
XLOC_004958-qpcr-R	AGCTGGAGGTGGCACCTTG
XLOC_004958-RT-F	TCCAGCTTTCTACTGCCTCC
XLOC_004958-RT-R	GGGAGGGAGCTTTATTTGGGA
XLOC_004958-GT-F	ACTAGCCAGCTGGTGGTTAC
XLOC_004958-GT-R	GGTTCTGTTCTTACTGCAGC

Supplementary Note. 1 Software used in the present study

No	Name	Version	Source code
1	GENCODE[3]	vM20	https://www.gencodegenes.org/
2	CPAT[4]	v2.2.0	http://lilab.research.bcm.edu/cpat/index.php
3	BLASTP[5]	v2.9.0	https://blast.ncbi.nlm.nih.gov/Blast.cgi
4	PFAM-A[6]	v31.0	http://pfam.xfam.org/
5	hmmer[7]	v3.2.1	http://www.hmmer.org/
6	IGV[8]	v2.4.4	http://software.broadinstitute.org/software/igv/download
7	salmon[9]	v0.10.0	https://github.com/COMBINE-lab/salmon
8	SUPPA2[10]	v2.2.1	https://github.com/comprna/SUPPA
9	SNPsplit	v0.3.4	https://github.com/FelixKrueger/SNPsplit
10	GMAP[11]	v2019-03-04	http://research-pub.gene.com/gmap/
11	cDNA_Cupcake	v6.6	https://github.com/Magdoll/cDNA_Cupcake
12	Cufflinks[12]	v2.2.1	http://cole-trapnell-lab.github.io/cufflinks/install/
13	FastQC	v0.11.8	http://www.bioinformatics.babraham.ac.uk/projects/fastqc/
14	TrimGalore	v0.6.1	http://www.bioinformatics.babraham.ac.uk/projects/trim_galore/
15	STAR[13]	v2.5.0a	https://github.com/alexdobin/STAR
16	StringTie[14]	v1.3.3b	https://github.com/gpertea/stringtie
17	TRANSDECODER[15]	v5.5.0	https://github.com/TransDecoder/TransDecoder
18	UniProt[16]	release 2019_08	https://www.uniprot.org/
19	STRING[17]	v11.0	https://string-db.org/
20	bigWigAverageOverBed	v2	http://hgdownload.soe.ucsc.edu/admin/exe/linux.x86_64/
21	MACS2[18]	v2.0.10.20131216	https://github.com/taoliu/MACS/
22	PANTHER[19]	v14.1	http://pantherdb.org/
23	SMRTlink	v6.0	https://www.pacb.com/support/software-downloads/
24	DEseq2[20]	V1.20.0	http://www.bioconductor.org/packages/release/bioc/html/DESeq2.html

Supplementary References

1. Gahurova L, *et al.* Transcription and chromatin determinants of de novo DNA methylation timing in oocytes. *Epigenetics Chromatin* **10**, 25 (2017).

2. Lloret-Llinares M, *et al.* The RNA exosome contributes to gene expression regulation during stem cell differentiation. *Nucleic Acids Res* **46**, 11502-11513 (2018).

3. Frankish A, *et al.* GENCODE reference annotation for the human and mouse genomes. *Nucleic Acids Res* **47**, D766-D773 (2019).

4. Wang L, Park HJ, Dasari S, Wang S, Kocher JP, Li W. CPAT: Coding-Potential Assessment Tool using an alignment-free logistic regression model. *Nucleic Acids Res* **41**, e74 (2013).

5. Altschul SF, *et al.* Gapped BLAST and PSI-BLAST: a new generation of protein database search programs. *Nucleic Acids Res* **25**, 3389-3402 (1997).

6. Finn RD, *et al.* The Pfam protein families database: towards a more sustainable future. *Nucleic Acids Res* **44**, D279-285 (2016).

7. Eddy SR. Accelerated Profile HMM Searches. *PLoS Comput Biol* **7**, e1002195 (2011).

8. Robinson JT, Thorvaldsdottir H, Wenger AM, Zehir A, Mesirov JP. Variant Review with the Integrative Genomics Viewer. *Cancer Res* **77**, e31-e34 (2017).

9. Patro R, Duggal G, Love MI, Irizarry RA, Kingsford C. Salmon provides fast and bias-aware quantification of transcript expression. *Nat Methods* **14**, 417-419 (2017).

10. Trincado JL, *et al.* SUPPA2: fast, accurate, and uncertainty-aware differential splicing analysis across multiple conditions. *Genome Biol* **19**, 40 (2018).

11. Wu TD, Watanabe CK. GMAP: a genomic mapping and alignment program for mRNA and EST sequences. *Bioinformatics* **21**, 1859-1875 (2005).

12. Trapnell C, *et al.* Differential gene and transcript expression analysis of RNA-seq experiments with TopHat and Cufflinks. *Nat Protoc* **7**, 562-578 (2012).

13. Dobin A, *et al.* STAR: ultrafast universal RNA-seq aligner. *Bioinformatics* **29**, 15-21 (2013).

14. Pertea M, Kim D, Pertea GM, Leek JT, Salzberg SL. Transcript-level expression analysis of RNA-seq experiments with HISAT, StringTie and Ballgown. *Nat Protoc* **11**,

1650-1667 (2016).

15. Haas BJ*, et al.* De novo transcript sequence reconstruction from RNA-seq using the Trinity platform for reference generation and analysis. *Nat Protoc* **8**, 1494-1512 (2013).

16. UniProt C. UniProt: a worldwide hub of protein knowledge. *Nucleic Acids Res* **47**, D506-D515 (2019).

17. Szklarczyk D*, et al.* STRING v11: protein-protein association networks with increased coverage, supporting functional discovery in genome-wide experimental datasets. *Nucleic Acids Res* **47**, D607-D613 (2019).

18. Zhang Y*, et al.* Model-based analysis of ChIP-Seq (MACS). *Genome Biol* **9**, R137 (2008).

19. Mi H, Muruganujan A, Ebert D, Huang X, Thomas PD. PANTHER version 14: more genomes, a new PANTHER GO-slim and improvements in enrichment analysis tools. *Nucleic Acids Res* **47**, D419-D426 (2019).

20. Love MI, Huber W, Anders S. Moderated estimation of fold change and dispersion for RNA-seq data with DESeq2. *Genome Biol* **15**, 550 (2014).
