# Supplementary material for: High-resolution annotation of the mouse preimplantation embryo transcriptome using long-read sequencing
Source: Nat Commun. 2020 May 27;11:2653. doi: 10.1038/s41467-020-16444-w (PMC7253418; doi:10.1038/s41467-020-16444-w)
Supplement: Supplementary file 15 — Reporting Summary [file 41467_2020_16444_MOESM15_ESM.pdf]

nature research

Corresponding author(s): Wenjie Shu

Last updated by author(s): Apr 17, 2020

Reporting Summary

Nature Research wishes to improve the reproducibility of the work that we publish. This form provides structure for consistency and transparency in reporting. For further information on Nature Research policies, see Authors & Referees and the Editorial Policy Checklist .

Statistics

For all statistical analyses, confirm that the following items are present in the figure legend, table legend, main text, or Methods section.

n/a	Confirmed	
☐	☒	The exact sample size (*n*) for each experimental group/condition, given as a discrete number and unit of measurement
☐	☒	A statement on whether measurements were taken from distinct samples or whether the same sample was measured repeatedly
☐	☒	The statistical test(s) used AND whether they are one- or two-sided<br>*Only common tests should be described solely by name; describe more complex techniques in the Methods section.*
☐	☒	A description of all covariates tested
☐	☒	A description of any assumptions or corrections, such as tests of normality and adjustment for multiple comparisons
☐	☒	A full description of the statistical parameters including central tendency (e.g. means) or other basic estimates (e.g. regression coefficient) AND variation (e.g. standard deviation) or associated estimates of uncertainty (e.g. confidence intervals)
☐	☒	For null hypothesis testing, the test statistic (e.g. *F*, *t*, *r*) with confidence intervals, effect sizes, degrees of freedom and *P* value noted<br>*Give P values as exact values whenever suitable.*
☒	☐	For Bayesian analysis, information on the choice of priors and Markov chain Monte Carlo settings
☒	☐	For hierarchical and complex designs, identification of the appropriate level for tests and full reporting of outcomes
☐	☒	Estimates of effect sizes (e.g. Cohen's *d*, Pearson's *r*), indicating how they were calculated

Our web collection on statistics for biologists contains articles on many of the points above.

Software and code

Policy information about availability of computer code

Data collection	GENCODE vM22,Cufflinks v2.2.1,UniProt release2019_08
Data analysis	CPAT v2.2.0, BLASTP v2.9.0, PFAM-A v31.0, IGV v2.4.4, salmon v0.10.0, SUPPA2 v2.2.1, SNPsplit v0.3.4, GMAP v2019-03-04, cDNA_Cupcake v6.6, FastQC v0.11.8, TrimGalore v0.6.1, STAR v2.5.0a, StringTie v.1.3.3b, TRANSDECODER v5.5.0, hmmer v3.2.1, STRING v11.0, bigWigAverageOverBed v2, MACS2 v2.0.10.20131216 PANTHER v14.1 SMRTlink v6.0, DESeq2 v1.20.0

For manuscripts utilizing custom algorithms or software that are central to the research but not yet described in published literature, software must be made available to editors/reviewers. We strongly encourage code deposition in a community repository (e.g. GitHub). See the Nature Research guidelines for submitting code & software for further information.

Data

Policy information about availability of data

All manuscripts must include a data availability statement. This statement should provide the following information, where applicable:

- Accession codes, unique identifiers, or web links for publicly available datasets
- A list of figures that have associated raw data
- A description of any restrictions on data availability

The long-read and short-read raw sequencing data were deposited at SRP: ‘SRP225196 [https://www.ncbi.nlm.nih.gov/sra?term=SRP225196]’. The processed data including transcriptome annotation and expression level matrix were deposited at GEO: ‘GSE138760 [https://www.ncbi.nlm.nih.gov/geo/query/acc.cgi?acc=GSE138760]’. CAGE data are obtained from ‘GSM3188176[https://www.ncbi.nlm.nih.gov/geo/query/acc.cgi?acc=GSM3188176]’, ‘GSM3188177[https://www.ncbi.nlm.nih.gov/geo/query/acc.cgi?acc=GSM3188177]’, ‘GSM3317700[https://www.ncbi.nlm.nih.gov/geo/query/acc.cgi?acc=GSM3317700]’, ‘GSM3317701 [https://www.ncbi.nlm.nih.gov/geo/query/acc.cgi?acc=GSM3317701]’. H3K4med data are obtained from ‘GSE73952[https://www.ncbi.nlm.nih.gov/geo/query/acc.cgi?acc=GSE73952]’. Custom oocyte transcriptome annotation are obtained from ‘GSE70116[https://www.ncbi.nlm.nih.gov/geo/query/acc.cgi?acc=GSE70116]’. The source data underlying Figure 1b-f; Figure 2a-d; Figure 3b-k; Figure 4a-g; Figure 5b, f, g; and Supplementary Figures 1-9 are provided as a Source Data file.

Field-specific reporting

Please select the one below that is the best fit for your research. If you are not sure, read the appropriate sections before making your selection.

- [x] Life sciences
- [ ] Behavioural & social sciences
- [ ] Ecological, evolutionary & environmental sciences

For a reference copy of the document with all sections, see nature.com/documents/nr-reporting-summary-flat.pdf

Life sciences study design

All studies must disclose on these points even when the disclosure is negative.

Sample size	We did not predetermined the sample size. Two batches of samples at each stage were collected. Pooled embryos at each stage from one batch, including 150 oocytes, 150 1-cell embryos, 100 2-cell embryos, 50 4-cell embryos, 25 8-cell embryos, 20 blastocysts and bulk sperms, were collected. These sample sizes are sufficient for RNA reverse transcription and amplification.
Data exclusions	No data were excluded from the analyses.
Replication	We carried out our experiments at least two times.
Randomization	Experimental groups were defined by experimental conditions. Within an experimental condition, the allocation was random.
Blinding	The investigators were blinded to group allocation during data collection and analysis. All experimental procedures and quantification of results were done by two independent researchers.

Reporting for specific materials, systems and methods

We require information from authors about some types of materials, experimental systems and methods used in many studies. Here, indicate whether each material, system or method listed is relevant to your study. If you are not sure if a list item applies to your research, read the appropriate section before selecting a response.

Materials & experimental systems

n/a	Involved in the study	
[x]	[ ]	Antibodies
[x]	[ ]	Eukaryotic cell lines
[x]	[ ]	Palaeontology
[ ]	[x]	Animals and other organisms
[x]	[ ]	Human research participants
[x]	[ ]	Clinical data

Methods

n/a	Involved in the study	
[x]	[ ]	ChIP-seq
[x]	[ ]	Flow cytometry
[x]	[ ]	MRI-based neuroimaging

Animals and other organisms

Policy information about studies involving animals; ARRIVE guidelines recommended for reporting animal research

Laboratory animals	house mouse, C57BL/6J (female) x DBA/2 (male) , female mice are 5 weeks old, male mice are 8 weeks old. Mice were maintained in an Assessment and Accreditation of Laboratory Animal Care credited specific pathogen free facility under a 12 h dark-light cycle. Ambient temperature is 20℃, relative humidity is 50%.
Wild animals	This study did not involve wild animals.
Field-collected samples	This study did not involve field-collected samples.
Ethics oversight	All experiments involving mice were approved by the Animal Care and Use Committee of the Institute of Neuroscience, Chinese Academy of Sciences, Shanghai, China.

Note that full information on the approval of the study protocol must also be provided in the manuscript.
