# Supplementary material for: High-resolution annotation of the mouse preimplantation embryo transcriptome using long-read sequencing
Source: Nat Commun. 2020 May 27;11:2653. doi: 10.1038/s41467-020-16444-w (PMC7253418; doi:10.1038/s41467-020-16444-w)
Supplement: Supplementary file 16 — Description of Additional Supplementary Files [file 41467_2020_16444_MOESM16_ESM.pdf]

Title: Supplementary Data 1:

Description: Novel isoforms and novel genes of merged transcripts identified by long-read sequencing. Transcripts are shown in gtf format.

Title: Supplementary Data 2:

Description: Novel transcripts with classical TSSs, novel TSSs or novel gene TSSs. Transcripts ID and TPM are shown in sheets. TPM was calculated using salmon.

Title: Supplementary Data 3:

Description: Novel transcripts with high-confidence novel TSSs or novel gene TSSs. Transcripts ID and TPM are shown in sheets. TPM was calculated using salmon.

Title: Supplementary Data 4:

Description: Novel transcripts with annotation of full-length reads support, H3K4me3 tag support, and CAGE-tag support.

Title: Supplementary Data 5:

Description: PCR amplification and Sanger sequencing of 10 novel genes

Title: Supplementary Data 6:

Description: PCR amplification and Sanger sequencing of 10 novel isoforms.

Title: Supplementary Data 7:

Description: Alternative splicing events identified from long-read transcripts across seven stages using SUPPA2.

Title: Supplementary Data 8:

Description: FPKM of splicing factors across six stages. The SFs with expression pattern of ZGA are shown in first column. *Padj* values represent the significance probability of 1-cell to 2-cell transition and are calculated using DEseq2 with wald

significance tests (adjusted for multiple testing using the Benjamini-Hochberg method).

Title: Supplementary Data 9:

Description: Differential alternative splicing events identified in consecutive stages using SUPPA2. *P-value* are calculated using an empirical cumulative density function (adjusted for multiple testing using the Benjamini-Hochberg method).

Title: Supplementary Data 10:

Description: TPM of novel ZGA transcripts across seven stages.

Title: Supplementary Data 11:

Description: Novel transcripts with remarkably upregulated during 1-cell to 2-cell transition in expression. Novel isoforms or novel genes with TPM of oocyte ≤ 1, TPM of 1-cell > 0.1, TPM of 2-cell > 1, 2-cell/1-cell > 2, 2-cell–1-cell > 10 and *Padj* value < 0.05 are presented. *Padj* values represent the significance probability of 1-cell to 2-cell transition and are calculated using DEseq2 with wald significance tests (adjusted for multiple testing using the Benjamini-Hochberg method).

Title: Supplementary Data 12:

Description: TPM of transcripts calculated with augmented transcriptome across seven stages using Salmon.
